# Chronic Exposure to Sodium Fluoride Triggers Oxidative Biochemistry Misbalance in Mice: Effects on Peripheral Blood Circulation

**DOI:** 10.1155/2018/8379123

**Published:** 2018-08-27

**Authors:** Giza Hellen Nonato Miranda, Bruno Alexandre Quadros Gomes, Leonardo Oliveira Bittencourt, Walessa Alana Bragança Aragão, Lygia Sega Nogueira, Aline Salgado Dionizio, Marília Afonso Rabelo Buzalaf, Marta Chagas Monteiro, Rafael Rodrigues Lima

**Affiliations:** ^1^Laboratory of Functional and Structural Biology, Institute of Biological Sciences, Federal University of Pará, Belém, PA, Brazil; ^2^Laboratory of Clinical Immunology and Oxidative Stress, Pharmacy Faculty, Institute of Health Science, Federal University of Pará, Belém, PA, Brazil; ^3^Department of Biological Sciences, Bauru Dental School, University of São Paulo, Bauru, São Paulo, SP, Brazil

## Abstract

The excessive fluoride (F) exposure is associated with damage to cellular processes of different tissue types, due to changes in enzymatic metabolism and breakdown of redox balance. However, few studies evaluate doses of F compatible with human consumption. Thus, this study evaluated the effects of chronic exposure to sodium fluoride (NaF) on peripheral blood of mice from the evaluation of biochemical parameters. The animals were divided into three groups (*n* = 10) and received three concentrations of NaF in the drinking water for 60 days: 0 mg/L F, 10 mg/L F, and 50 mg/L F. The blood was then collected for trolox equivalent antioxidant capacity (TEAC), thiobarbituric acid reactive substances (TBARS), concentrations of nitric oxide (NO), superoxide dismutase (SOD), catalase (CAT), and reduced glutathione (GSH). The results showed that doses of 10 mg/L F and 50 mg/L F were able to increase TBARS concentration and decrease NO levels and CAT activity in the blood, but there was no statistical difference for SOD levels. The 50 mg/L F group showed an increase in TEAC levels and a decrease in the GSH content when compared to the control group. In this way, oxidative changes in blood from chronic exposure to F, especially at the highest dose, indicate that F may be a toxic agent and, therefore, the long-term exposure to excessive doses should be avoided.

## 1. Introduction

Fluoride is a negatively charged nonmetallic halogen that can be naturally available in the soil, rocks, and water [1]. Fluoride can also be artificially added to the drinking water, which constitutes, together with fluoridated dental products, the main source of fluoride for human consumption [1]. Small fluoride concentrations have therapeutical action against dental caries. However, exposure to high doses from water ingestion and the use of fluoride toothpastes or fluoride-rich diets increases the body burden of this ion [2].

Water fluoridation initiated in the United States in 1945 and is currently practiced in approximately 25 countries around the world [3]. This strategy has been recognized as one of the most effective ways of ensuring community-wide exposure to the effects of fluoride on caries prevention [4]. Despite previous studies attesting the safety of community water fluoridation [5], from an ethical point of view, fluoridation is configured as an intervention for environmental level, in which the individual conformity is not questioned. Thus, the community water fluoridation a medication method without individual consent or choice [6].

The fluoride can act as an enzyme inhibitor, due to its strong electronegativity. Thus, it forms ions in solution and the main toxic effect of fluoride derives from its interaction with enzymes [7]. On the other hand, fluoride can also stimulate the enzymatic activity through mechanisms dependent on time, concentration, and cell type [7]. For example, fluoride at lower concentrations (*μ*M) acts as a stimulator and promotes cell proliferation, while at higher concentrations (mM), it inhibits enzyme action, including phosphatases [7, 8]. In addition, high doses of ingested fluoride might damage several biological systems, including the central nervous system [9], reduction of splenic and humoral cell immunity [10], dysfunction of the male reproductive system [11], and liver damage [12, 13]. Evidence in animal models suggests that fluoride concentrations above 5 mg/L in drinking water can modify cellular processes such as respiration and metabolism, thus leading to oxidative stress [7].

After ingestion, fluoride is absorbed from the gastrointestinal tract, circulates in the organism and is taken up mainly by mineralized tissues and to a lower extent by soft tissues. The remaining amount is excreted mainly in the urine [14]. After 10 minutes from the fluoride absorption, its plasmatic concentration increases, reaching the maximum peak at 60 minutes. The return to baseline levels is achieved within 11 to 15 hours; then, fluoride is rapidly deposited in the skeleton or excreted by the kidneys [15]. Once fluoride is incorporated into the bone, especially in the crystal structures or the bone matrix, it can be slowly removed with a half-life of 120 weeks for adults and 70 weeks for children [16]. Previous studies with rats chronically exposed to fluoride demonstrate the impairment in essential organs on metabolism and excretion processes, as liver and kidney, by oxidative stress, which is a critical cell damage [13, 17].

Although fluoride is absorbed largely by the mineralized extracellular matrix in calcified tissues, absorbed fluoride can lead to mitochondrial dysfunction, DNA damage, and lipid peroxidation in cells through the production of reactive oxygen species (ROS) [18, 19].

The association between absorbed fluoride and changes in oxidative parameters is an important indicator of the toxic potential of fluoride on cellular mechanisms. The great importance of evaluating oxidative stress markers such as MDA in the peripheral blood is that this site is a useful source of biomarkers, as it is easily obtained and minimally invasive. Therefore, alterations in oxidative parameters can be detected in individuals exposed to compounds, even at low doses, and can distinguish them from individuals not exposed to these compounds or their metabolites. Accordingly, many studies show that biomarkers are preferentially quantified in accessible biological matrices (e.g., urine and blood). After fluoride reaches the systemic blood circulation, multiple organs are affected by exposure to the substance, but it is not clear yet by which mechanisms fluoride leads to systemic dysfunction. Thus, this study aimed at assessing the effect of fluoride exposure in levels similar to the ones found in areas of artificial water fluoridation and in areas of endemic fluorosis in blood oxidative processes, investigating that even small concentrations can trigger mechanisms that damage the body.

## 2. Material and Methods

### 2.1. Animals and Treatment

Thirty male Swiss albino mice (*Mus musculus* with 21 days of age, 30 ± 10 g) were randomly divided into three groups (*n* = 10 per group). The animals were maintained in polypropylene cages (5 per cage), with *ad libitum* access to food and water, controlled temperature, and humidity and with regular light/dark cycles. The mice exposed to fluoride received deionized water containing 10 or 50 mg/L of fluoride as sodium fluoride (NaF; Sigma Chemical, USA) during 60 days. The nonexposed group (control) received deionized water during the same period. The experimental protocol (register 57-2015) was approved by the Ethics Committee for Animal Experiments of Federal University of Pará, Brazil. At the end of the experiment 60 days, the blood samples were collected from the animals through intracardiac puncture. Then, the blood samples were transferred to tubes for further methodological steps, as described below.

### 2.2. Sample Preparation

Blood samples were collected in tubes containing 50 *μ*L of 5% ethylenediamine tetraacetic acid (EDTA) and centrifuged 3000 rpm for 10 minutes. After centrifugation, plasma and red blood cells were collected and stored separately in microtubes, proceeding the storage of plasma at −80°C and washing of red blood cell suspensions with 0.9% saline solution with consecutive centrifugation at 2500 rpm for 10 minutes (procedure was repeated twice) to obtain 50% red blood cells, ready for frozen storage at −80°C, for further enzymatic analysis determination. Plasma samples were analyzed for thiobarbituric acid reactive substances (TBARS), trolox equivalent antioxidant capacity (TEAC), nitric oxide (NO) concentration, and fluoride concentration.

### 2.3. Fluoride Analysis

Fluoride concentrations in plasma were determined according to Whitford and Taves [20, 21]. This method uses a specific ion fluoride electrode (Orion Research, Model 9409) and a miniature calomel electrode (Accumet #13-620-79), both coupled to a potentiometer (Orion Research, Modelo EA 940). Firstly, plasma was prediffused to remove CO_2_. Fluoride concentrations in plasma were determined after acid-hexamethyldisiloxane (HMDS)-facilitated microdiffusion. Fluoride standards (0.0048 and 0.19 *μ*g F) were prepared in triplicate and diffused similarly as the samples. Nondiffused standards prepared had the same concentration as the diffused ones standards. The millivoltage (mV) readings were converted to *μ*g F using Excel (Microsoft). The coefficient adopted to standard curve was *r* ≥ 0.99. The comparison of the mV readings showed a complete extraction of fluoride (recovery higher than 95%). Fluoride concentration was expressed in *μ*g/mL.

### 2.4. Oxidative Biochemistry Assays

#### 2.4.1. Nitric Oxide (NO) Concentrations

The NO was quantified as nitrate concentration based on the Griess method [22]. Nitrate concentration in plasma samples was converted to nitrite by nitrate reductase. Briefly, 100 *μ*L of samples was incubated with Griess reagent (100 *μ*L) for 10 minutes at 37°C. Absorbance was measured in a microplate reader (Spectra Max 250, Molecular Devices, Menlo Park, CA, USA) at 550 nm. Nitrate concentration was determined expressed in *μ*mol/L.

#### 2.4.2. Measurement of Thiobarbituric Acid Reactive Substances

Lipid peroxidation (LPO) was measured by determining the thiobarbituric acid reactive substances (TBARS) as described by Kohn and Liversedge [23] and modified by Percário [24]. The malondialdehyde (MDA) produced after the lipid peroxidation process reacts with thiobarbituric acid (TBA) and generates chromophore substance. Briefly, 1 mL of 10 nM TBA was added to 100 *μ*L of samples, following incubation for 1 h at 94°C. Samples were cooled, n-butanol (4 mL) was added in each sample, and then samples were homogenized and centrifuged at 2500 rpm for 10 minutes. The organic phase (3 mL) was spectrophotometrically read at 535 nm. The concentration of TBARS was expressed in *μ*mol/L.

#### 2.4.3. Measurement of Trolox Equivalent Antioxidant Capacity (TEAC)

The method used to analyze TEAC levels is described by Ruffino et al. [25]. This is a nonspecific method for the determination of the total antioxidant activity of body fluids [26]. In this assay, 2,2′-azino-bis(3-ethylbenzothiazoline)-6-sulfonic acid (ABTS; 7 mM) was incubated by adding potassium persulfate (2.45 mM) at room temperature during 16 h to produce ABTS^+^ radical. The work solution was prepared from ABTS^+^ radical in phosphate basic saline (PBS) solution (pH 7.2) until absorbance of 0.7 ± 0.02 at 734 nm. Subsequently, 30 *μ*L plasma or trolox standards (standard curve) were added to 2970 *μ*L of ABTS solution, and absorbance was acquired after 5 minutes. Absorbances were measured in triplicate and calculated following a standard curve with trolox [27] as a standard. The total antioxidant capacity in plasma was expressed in *μ*mol/L.

#### 2.4.4. Catalase Activity (CAT)

The CAT enzyme activity was determined according to the method described by Aebi [28]. Erythrocytes were hemolyzed in milli-Q water (1 : 3) and then diluted in Tris-buffer solution (0.1 M Tris HCl/5 mM EDTA; pH 8.0). The hydrogen peroxide (H_2_O_2_) degradation was registered after the addition of 900 *μ*L reaction solution (1 M Tris HCl, 30% H_2_O_2_, and ultrapure water; pH 8) in 100 *μ*L of hemolyzed solution. The CAT activity was defined as activity necessary to degrade 1 mol of H_2_O_2_ during 60 seconds and expressed in U/mg of protein.

#### 2.4.5. Superoxide Dismutase Activity (SOD)

The SOD activity was determined by following the modified method of McCord and Fridowich [29]. This method evaluates the capacity of SOD to convert radical superoxide (O_2_^−^) to hydrogen peroxide (H_2_O_2_) and oxygen (O_2_). First, the erythrocyte suspensions were hemolyzed in milli-Q water. The total SOD activity in red blood cells was determined by reducing the cytochrome c (cyt C) (0.075 mM) through anion superoxide generated by xanthine/oxidase xanthine system in a spectrophotometer at a wavelength of 550 nm [30]. The results were expressed in nmol/mL.

#### 2.4.6. Reduced Glutathione Content Measurements (GSH)

The GSH level measurements were determined by using a modified Ellman method [31]. First, the red blood cells were hemolyzed in cold distilled water. An aliquot (20 *μ*L) from hemolyzed was added in a tube containing distilled water (20 *μ*L) and PBS-EDTA buffer solution pH 8.0 (3 mL) to carry out the first measurement. Then, 5,5′-dithiobis(2-nitrobenzoic acid) (DTNB; 0.47mmol) was added to solution, and another measurement was carried out after 3 minutes. The GSH concentration was expressed as *μ*g/mL.

### 2.5. Statistical Analysis

Data were expressed as mean ± standard deviation for each fluoride levels and percentage of the control ± standard deviation for oxidative biochemistry assays. To calculate the standard data distribution, the normality Shapiro-Wilk test was performed. The data passed on normality and were analyzed by one-way ANOVA followed by Tukey's test. The significance level adopted was *p* < 0.05. The software GraphPad Prism 5.0 (San Diego, CA, EUA) was used for all analysis.

## 3. Results

### 3.1. Levels of Fluoride in Plasma after 60-Day Exposure

**Figure 1:**
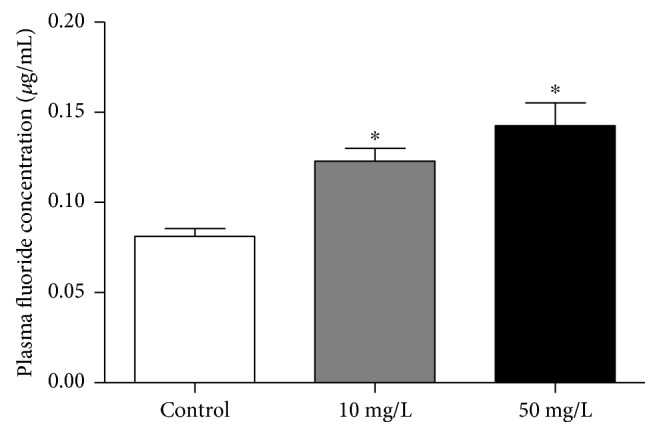
Analysis of plasma fluoride concentration. The graph shows the fluoride concentration in the plasma of mice in *μ*g/ml after 60 days of deionized water (control group), 10 mg/L fluoride water and 50 mg/L fluoridated water. One-way ANOVA followed by Tukey's test, *p* < 0.05. ^∗^Statistical difference in relation to the control.

After 60-day exposure, the fluoride concentrations in the 10 mg/L NaF treatment (0.122 ± 0.0071) and 50 mg/L NaF treatment (0.142 ± 0.0127) were statistically higher when compared to control group (0.081 *μ*g/ml ± 0.0044; *p* = 0.0003) (Figure 1).

### 3.2. Oxidation Parameters in Plasma and Red Blood Cell Samples

**Figure 2:**
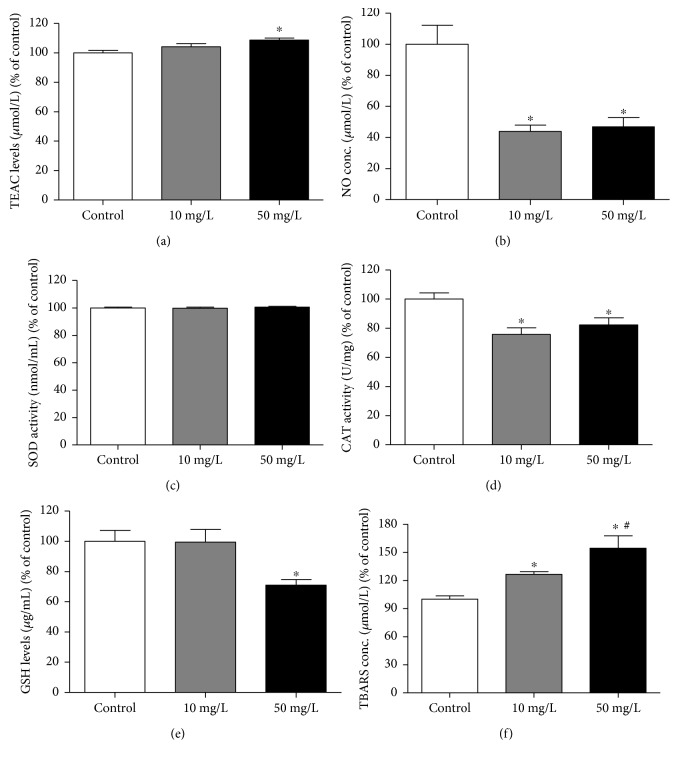
Evaluation of oxidative biochemistry in blood. The graphs represent, as a percentage of the control, the results of oxidation biochemistry in the groups that received deionized water, 10 mg/L fluoride water and 50 mg/L fluoride water after the experimental period (60 days). (a) TEAC levels, (b) NO concentration, (c) SOD activity, (d) CAT activity, (e) GSH levels, and (f) TBARS concentration. One-way ANOVA followed by Tukey's test, *p* < 0.05. ^∗^Statistical difference in relation to the control; ^#^statistical difference in relation to the 10 mg/L group.

As observed in Figure 2, the chronic fluoride exposure also altered oxidative parameters in plasma and red blood cells. The highest fluoride concentration (50 mg/L F) increased TEAC (*p* = 0.01) and TBARS (*p* = 0.0001) levels and caused a decrease of GSH levels (*p* = 0.004) and NO concentration (*p* = 0.001) in the plasma of exposed animals compared to control. Moreover, animals chronically exposed to 10 mg/L F of fluoride also showed significantly lower levels of NO (*p* = 0.001), significantly decreased CAT activity (*p* = 0.002), and a significant increase of TBARS levels (*p* = 0.0001) in blood samples. In contrast, SOD activity did not show statistical difference among the groups (*p* = 0.79).

## 4. Discussion

In the present study, fluoride exposure significantly increased plasma fluoride concentrations. Moreover, chronic fluoride exposure induced biochemical alterations in the peripheral blood of mice, such as increased lipid peroxidation levels and decrease of the CAT activity and NO levels.

The fluoride exposure doses used in our study (10 and 50 mg/L) are often employed [32–34] and lead to a plasma fluoride levels in rodents similar to the ones found in humans consuming artificially fluoridated water or living in areas of endemic fluorosis, respectively [35]. It is important to note that F metabolism in rodents is 5–10 times faster than that in humans and the concentrations used in this investigation, 10 and 50 mg/L, correspond to 1-2 and 5–10 mg/L, respectively, for humans in the drinking water [35].

Once absorbed into the blood, fluoride is distributed rapidly throughout the body and is mainly retained in areas rich in calcium, such as bones and teeth (dentin and enamel). The fluorine with calcium forms calcium ionospheres that readily diffuse into the cell membrane [7]. Fluoride, mainly in the form of hydrogen fluoride (HF), is transported through the cell membrane by nonionic diffusion [36]. The main mechanism of fluoride toxicity in cells is associated with its ability to interact with enzymes; most often, the fluoride can lead to inhibition of enzymatic activity (e.g., phosphatases, GTPases, and ATPases). In addition, fluoride can also inhibit the protein secretion and/or synthesis involved in signaling pathways (mitogen-activated protein kinase (MAPK), p53, activator protein-1 (AP-1), and nuclear factor kappa B (NF-*κ*B) [37–39] and antioxidant enzymes (SOD, glutathione levels, and CAT) [40]. Thus, the inhibition of antioxidant enzymes results in the excessive production of ROS at the mitochondrial level, leading to cell damage. On the other hand, the fluoride at lower concentrations may stimulate enzymatic activity and promotes the increase of the cell proliferation and apoptosis because of the increase in proapoptotic proteins, such as caspase-3 and caspase-9 [41, 42]. Therefore, fluoride can also induce oxidative stress leading to the production of ROS, which triggers the release of cytochrome c from mitochondria into the cytosol and further activates caspase-3 leading to apoptotic cell death [41, 42].

In this regard, the organisms have a variety of antioxidant molecules and mechanisms that protect them against ROS, which include the enzymes SOD, CAT, and glutathione peroxidase (GSH-Px), and nonenzymatic antioxidants such as selenium and vitamins A, E, and C as well as compounds containing thiol groups [43]. Imbalance between ROS and antioxidant system characterizes an oxidative stress condition [44]. It is important to consider that high levels of oxidative damage may result not only from the increase in pro-oxidative species but also from failures in the repair and replacement system [44].

In our study, the enzymatic assays showed that chronic fluoride treatment did not alter SOD activity when compared to control. Similar results were observed in human and rabbits exposed to 5 mg/L of fluoride in drinking water for 6 months [45]. However, fluoride can also act as a competitive SOD inhibitor decreasing the enzyme activity after binding to its active site [46] or stimulating superoxide radical production which is substrate for SOD, which consequently increases enzyme activity [43, 47]. It is possible that the lack of alteration in SOD activity in the exposed groups is related to an equilibrium caused by compensatory mechanisms.

The CAT activity was significantly decreased upon treatment with fluoride, regardless of the dose. However, only the highest fluoride dose significantly decreased GSH levels compared to control, denoting that fluoride can act as inhibitor of enzymatic antioxidants (CAT) or nonenzymatic antioxidants (GSH). Several studies report reduced enzymatic activity after fluoride exposure, followed by oxidative damage [48–50]. Other studies show that fluoride reduces CAT activity and GSH levels, but not the activity of SOD activity in human erythrocytes [49] and in the liver of mice [51].

The total antioxidant capacity is commonly maintained by enzymatic and nonenzymatic systems, which reflect the compensatory capacity against external stimulus [52]. In our study, the TEAC levels showed a significant increase upon exposure to 50 mg/L fluoride, similar to blood samples of animals exposed to 50 mg/L fluoride for 12 months [52]. This result suggests a possible response of the organism through compensatory mechanisms against the biomolecular damage caused by fluoride. Changes in antioxidant responses result from excessive production of mitochondrial ROS, which damage cellular components, including membrane phospholipids that undergo lipid peroxidation, mitochondrial membrane depolarization, and apoptosis [53].

Studies show that treatment with antioxidants, such as ascorbic acid, tamarind seed coat extract, blackberry, and quercetin, prevented fluoride-induced changes such as increase of oxidant (reactive oxygen species generation, lipid peroxidation, protein carbonyl content, and NO) and inhibition of antioxidant (superoxide dismutase, catalase, glutathione peroxidase, and glutathione) parameters, suggesting that the major mode of action of fluoride is dependent of oxidative/nitrosative mechanism [53–55].

The oxidative stress, therefore, is characterized as an excess of ROS or decrease of antioxidant defenses that results in cellular macromolecule damage and changes cellular homeostasis [56, 57]. In this sense, fluoride exposure increases the generation of superoxide (O_2_^−^) and other reactive oxygen species (ROS), as well as fluoride-induced cytotoxicity with increased ROS generation, which may activate the sirtuin 1 (SIRT1)/autophagy pathway through JNK signaling, as an adaptive response that leads to cell protection [58]. These alterations can be measured by the increase in the peroxidation of the polyunsaturated fatty acids in the cell membranes, resulting in an increase of TBARS and MDA [49]. In our study, the animals chronically exposed to fluoride showed an increase of TBARS levels when compared to control. LPO is an important form to evaluate the levels of oxidative stress. The oxidation of unsaturated fatty acids, like those found in organelles and cell membranes, may lead to rearrangement of membrane composition or cell death process [59, 60]. In our study, we observed that MDA levels increased after fluoride exposure in a dose-dependent manner. In this way, we strongly believe that as the fluoride concentration increases, more antioxidant enzymes are impaired and, consequently, the MDA increases. In addition, considering that RBC have a high quantity of fatty acids, such as cholesterol, probably the RBC life spam and deformability may be impaired, once we found NO decrease and MDA increase [61, 62].

Several studies reported fluoride as an inducer of oxidative stress and modulator of intracellular redox homeostasis, lipid peroxidation, and protein carbonyl content [7, 49, 50]. These processes occur due to the ability to break hydrogen bonds in proteins (e.g., in the enzyme active center), as well as increasing the mitochondrial generation of free radicals resulting in oxidative stress, mitochondrial DNA degradation, and cell death [10, 63, 64].

In normal conditions, the superoxide anion produced by vascular walls is detoxified by the enzyme SOD into hydrogen peroxide (H_2_O_2_) that may be converted into inactive forms by Fenton's reaction or other enzymes, such as GSH-Px (before action on hydrogen peroxide) and CAT [65, 66]. However, a reduction of any of these enzymes may lead to an oxidative imbalance. In our study, we found that although SOD activity is not impaired by fluoride exposure, the subsequent enzymes of the detoxification process are reduced (CAT and GSH) [65]. Moreover, their inhibition seems to be dose-dependent, since GSH only decreased in the highest fluoride dose group. In this way, the increase of ROS may also drive to lipid peroxidation.

The importance of NO has been related to several biological processes, as inflammatory response, immunity, endothelial relaxation, and others [66, 67]. Clinically, it has been reported that lower levels of NO implicates on the prediction to several cardiovascular diseases, as arteriosclerosis and hypertension, due to its effects on vasculature [66, 68].

The production of nitric oxide in our experiments decreased significantly after fluoride exposure. NO is a homeostasis regulator [69], and its inactivation occurs through superoxide anion reaction producing peroxynitrite, a compound capable to cause oxidative damage to biomolecules, including proteins, lipids, and DNA [70]. In this regard, an *in vitro* study showed that NaF increased the release of cytochrome c (cyt C) from the mitochondria to the cytosol, as well as the levels of ADP, AMP, GDP, and Pi, but decreased ATP production. Persistent inhibition of these factors results in the induction of NO that inhibits mitochondrial respiration by decreasing the apparent affinity of cyt C for oxygen [7, 71, 72]. In addition, NO is able to react with superoxide anion (O_2_^−^) and to produce toxic substances, such as peroxynitrite or thiols and metal centers in proteins to form nitrosyl adducts [73]. NO also plays an autocrine function by modulating the deformability of red blood cells (RBC), thus favoring their passing through the capillaries and improving the microcirculation [74, 75]. In addition, an increased oxidative stress may reduce the NO bioavailability through an impairment of the NO synthesis and through the inactivation of the NO produced by transforming it into peroxynitrate/nitrite [76, 77]. These factors can interfere with disulfide bond formation and result in the accumulation of misfolded proteins in the endoplasmic reticulum (ER) causing ER stress and ROS production [73].

Changes in TBARS, NO, and TEAC, as well as CAT activity and GSH levels, especially in the group exposed to the highest dose of fluoride, indicate that this ion is a toxicant, inducing metabolic alterations in the blood and interacting with the antioxidant system in mice chronically exposed. Thus, exposure to excessive fluoride doses in the long term must be avoided.

## Data Availability

The data used to support the findings of this study are available from the corresponding author upon request.
